# Experimental Researches on Inanition; the Memoir to Which the Prize for Experimental Philosophy Was Adjudged by the Academy of Sciences in 1841

**Published:** 1844-04

**Authors:** 


### Art. IV.

*Recherches Expérimentales sur l'Inanition; Mémoire auquel l'Académie des Sciences a décerné, en* 1841, *le prix de Physiologie expérimentale.* Par Charles Chossat, m.d., Membre de plusieurs sociétés savantes. —*Paris*, 1843. 4to, pp. 202.

*Experimental Researches on Inanition; the Memoir to which the Prize for Experimental Physiology was adjudged by the Academy of Sciences in* 1841. By Charles Chossat, m.d. &c.—*Paris*, 1843.

Were we in a moralizing humour, we might take this Essay as a text for a disquisition on the question of the *right* of physiologists to subject other sentient beings by the score, or even (as in the present instance) by the hundred, to the severest tortures, without a sufficient probability of the deduction of some important practical inference, or of some scientific truth, which may hereafter, if not at present, be applicable to the benefit of the human race. But we must defer this subject until some future opportunity; simply contenting ourselves with the remark, that, while we regard the physiologist as justified in putting nature to the question by well-devised experiments, even on living animals in cases in which *no other* means appear likely to educe the desired results, we cannot but condemn the wholesale slaughter which is practised, apparently without the least compunction, by many continental physiologists,—too often, in mere wantonness as it would seem, without any distinct idea of the ends to be attained,—and, as frequently, for the mere *exhibition* of effects, which have been long since positively ascertained, and our knowledge of which is not made one whit more certain or definite by the repetition of the experiment. We hold it to be the bounden duty of the experimental physiologist not to subject living animals to pain, until he has, by anatomical study, by the observation of natural and morbid phenomena, and by the due consideration of the data supplied by *comparative* anatomy and physiology, made himself fully acquainted with all that these can teach; and thus enabled himself to plan his experiment in such a manner, that he may draw from the smallest number of these trials, involving the least possible amount of suffering, the information which he seeks. Let any candid inquirer compare the *results* obtained by Sir C. Bell and Dr. M. Hall,* on the one hand, with the amount of animal suffering which they inflicted; and then make a corresponding comparison between the results obtained by Magendie, Brachet, Longet, and other wholesale French vivisectors, and *their* sacrifice of animal life, and their infliction of torture,—not upon individuals, but upon thousands of sentient beings.

We cannot but include in our censure the author of the 'Experimental Researches' now before us; since it appears that the *ends* attained are utterly disproportioned to the *means* employed; as might, we think, have been predicted by any well-judging physiologist, had the nature and plan of the experiments been set before him. Moreover, the suffering was not for a few seconds or minutes only, as in the case of most well-devised physiological experiments, but was prolonged for days and weeks; being, in fact, nothing else than the *starvation* of many hundreds of unfortunate pigeons, turtle-doves, and other animals, to ascertain their loss of weight, and other facts, of which the greater number cannot be extended beyond the particular species which were the subjects of the experiments, and which, therefore, can serve but little for the advancement of Human Physiology. We shall endeavour to select the most interesting results; that our readers may derive what benefit they can from the investigation. We must premise, however, that many points of the *most general* importance, which would have occurred, we think, to any well-informed physiologist, seem to have been utterly neglected during the whole course of the inquiry; such, for instance, is the relative *activity* of the animals during the period of starvation, which is well known to have a most important influence on the duration of life under such circumstances.

The first series of experiments relates to the diurnal loss of weight, under a total privation of food and drink. This is usually greatest on the first day, as might be expected, from the discharge of the contents of the intestinal canal, without any corresponding ingestion of aliment. The diurnal loss then usually diminishes, until past the middle period during which life is protracted; and it then commonly undergoes a sudden and remarkable increase, especially within a day or two before death. Thus, in one of the pigeons which lived the longest, the diminution on the first day was nearly $14\frac{1}{2}$ grammes; on the second it was but $9\frac{1}{2}$; from this it decreased until the eleventh day, when it was but $6\frac{1}{2}$; but it then began to increase again, so as to be 9 grammes on the sixteenth day; 10 on the seventeenth; 12 on the eighteenth; and 14 on the nineteenth and last. In another instance, the daily loss was, $25\frac{1}{2}$ grammes on the first day; by the fourth it had diminished to 12; it then began to increase

* It is to be remembered that, in a great proportion of Dr. Hall's experiments, the *sensation* of the subjects of them is destroyed *in limine.*

again, so as to reach nearly 20 on the ninth day, and 32 on the tenth and last. By the comparison of these two cases with each other, it is evident that the shorter duration of life in the second is connected with the more rapid decrease in its weight; and the same result presents itself, when the results of numerous other experiments, recorded by M. Chossat, are compared together. We are left utterly in the dark, however, as to the most important influence likely to have caused this inequality, namely, the relative amount of muscular exertion employed by the different individuals. Had this been recorded, we should have had some important data, by which to judge of the doctrine, that the *waste* of the body is in proportion to the amount in which its different functions are exercised. The increase in the excretions during the last few days of life, is a very curious occurrence; and seems to indicate that the fabric is then undergoing more rapid disintegration,—a view which corresponds with the fact, that the bodies of persons that have died of starvation very early exhale a putrescent odour, which is even manifested before death; as if the solids and fluids were already subjected to those changes, which usually take place some time subsequently to dissolution. It is probable that the colliquative diarrhea, which so frequently manifests itself at the termination of exhausting diseases, is to be regarded as an analogous occurrence; and that it is rather the *effect* of the near approach of death, indicating that the organic structure cannot any longer hold together, than the cause of the fatal termination, as it is usually regarded.

It appears, from M. Chossat's experiments, that whether life during inanition be more or less prolonged, the total loss, compared with the original weight of the animal, is always nearly the same in the same species. The average, in warm-blooded animals, he states to be about 40 per cent.; but there is a considerable variation in this limit; for in two fowls it was nearly 53 per cent., whilst three guinea-pigs died when the loss had reached (on the average of all) 33 per cent. One of the circumstances which most influence the amount of sustainable loss of weight, is the quantity of fat previously accumulated; for it will be shown hereafter, that fat is almost entirely removed during inanition; and, consequently, those who previously possess most of it have most to lose during the process. This is the explanation of the greater proportional loss of weight in the two fowls, which were previously very plump, than in the pigeons and turtles, in which it was from 38 to 41 per cent.

It has been long known that *age* has a most important influence in modifying the prolongation of life during inanition; the life of the adult being usually extended much beyond that of the child or the aged person; and on this principle are founded the presumptions of survivorship in forensic medicine. We might have reasonably expected some important information on this head from M. Chossat's researches; but we are altogether disappointed, in consequence, it would seem, of his not being able to ascertain the relative ages of the subjects of his experiments. He endeavours to form an estimate of their respective ages by their relative weights, classing his turtle-doves as *young*, *middle-aged*, or *adult*, according as their weights were beneath 120 grammes, from 120 to 160, or above 160. We need hardly stop to point out how very fallacious must be any such criterion of age; since there must be differences in weight among individuals of the same age that would cause one of them to be placed as young, and another as adult. But the result is interesting, when viewed merely as bearing upon the question of the duration of life, and the loss of substance during inanition, in animals of different weights.

| | Weight of the Body. | | Loss of the Body. | | | |
|---|---|---|---|---|---|---|
| | *Weight at commencement.* | *Weight at death.* | *Entire absolute loss.* | *Proportional loss in* 1000 *parts.* | *Daily proportional loss.* | *Duration of life.* |
| | Gram. | Gram. | Gram. | | | |
| *a.* Young . | 110·42 | 82·84 | 27·58 | 0·250 | 0·081 | 3·07 |
| *b.* Mid.-aged | 143·62 | 91·60 | 52·02 | 0·362 | 0·059 | 6·12 |
| *c.* Old . | 189·36 | 101·61 | 87·75 | 0·463 | 0·035 | 13·36 |

It will be perceived, from this comparison, that the entire *actual* and *proportional* loss were both much greater in the heaviest animals; but that the diurnal loss was much the most rapid in the lightest; and the general result may probably be received as confirmatory of the previously-received opinion—the difference in the duration of life being much greater than could be accounted for on the previous *condition* of the animal simply, and evidently resulting in great part from the more rapid *waste* which takes place in the young animal.

Another series of experiments of the same kind was made upon frogs, the duration of whose life, when they were placed in pure water frequently renewed, but without any food whatever, varied from six to sixteen months. Notwithstanding this great difference in regard to time, the actual amount of loss sustained was very nearly the same, in proportion to the previous weight of the body, as in birds; being usually from 33 to 48 per cent., although reaching, in two cases, to 54 and 58 per cent. Other experiments were made upon various species of cold-blooded animals, with the same general result; which, when compared with that obtained from all the experiments on warm-blooded animals, becomes additionally striking.

| | *Average Duration of Life.* | Proportional Loss of Weight. | |
|---|---|---|---|
| | | *Daily loss in* 1000 *parts.* | *Entire loss in* 1000 *parts.* |
| Warm-blooded Animals ...... | 9·68 days. | 0·0420 | 0·397 |
| Cold-blooded Animals ...... | 226·0 ,, | 9·0021 | 0·404 |

The duration of life, in the second class of animals is seen to be *twenty-three* times that of the first; but, on the other hand, the proportional daily loss is no more than one twentieth part, in the cold-blooded animals, of its amount in the warm-blooded: so that the entire loss comes to be very nearly the same; or, in other words, the cold-blooded animal dies at the same amount of *inanition* as the warm-blooded; but is longer in reaching the limit.

The object of the next series of experiments was to determine the result of *insufficient* alimentation. A number of turtle-doves were supplied with limited quantities of corn, but with water at discretion; and two very interesting results presented themselves. In the first place, the amount of loss was almost exactly the same as in the case of complete abstinence; but life was prolonged for about double the length of time. Secondly, it appeared that in scarcely any instance was the whole amount of food that the birds were allowed to take actually digested; a part of it being rejected by vomiting, or passing off by diarrhea, or accumulating in the crops. It seems that the vital power was not sufficient to furnish the requisite supply of gastric fluid, when the body began to be enfeebled by insufficient nutrition; or, perhaps we might well say, the materials of the gastric fluid were wanting. We need scarcely point out the very important practical applications of this principle. The loathing of food, often manifested by those who have been subjected to the influence of an insufficient diet-scale in our prisons and poor-houses, has been unjustly set down to caprice or obstinacy, and punished accordingly; whereas it is actually a proof of the deficiency of the supply, which, it might be imagined, would have been voraciously devoured if this were really the case.

The next subject considered is the influence of deprivation of water; and here we encounter so many sources of difference, arising from the variations which exist in the demand for water among the several tribes of animals, that it is impossible to arrive at any *general* conclusion. Thus, birds naturally drink but very little; for the amount of their loss of fluid by excretion is very small, and a little, therefore, serves to replace it.*

When a number of pigeons and turtle-doves were totally deprived of solid aliment, and were freely supplied with water, it was found that they did not drink above one fifth or one sixth of their natural quantity. There was no decided difference in the duration of life, between the pigeons that were deprived of liquid as well as of solid aliment, and those which were freely supplied with the former; but in the turtle-doves, the duration of life was more than *twice as great* in those which were deprived of water, as in those which were freely supplied with it. This unexpected result M. Chossat explains by saying, that the weight of the former series was greater by a fourth than that of the latter; but on this we would remark, that by neglecting to select animals for comparison which were as nearly as possible in the same circumstances, M. Chossat appears to us to have shown great carelessness—we would say culpability; for, by this omission, a number of birds were subjected to the pangs of a lingering death by starvation, to produce a result altogether valueless. The rabbit was the only mammal on which an experiment of this kind was made; and only a small number were subjected to it. The duration of life appears to have been sensibly greater, however, when water was supplied than when it was withheld, being nearly thirteen days in one case and ten and half in the other; and the difference would probably have been more striking, had animals of the same weight been selected for comparison, for M. Chossat remarks that the animals which lived the longest

* We are not aware that any attempt has ever been made to assign the *rationale* of this. The following may be thought an approach to it. Of the quantity of fluid which *we* take in, a large proportion is thrown off by cutaneous and pulmonary exhalation, serving to keep down the temperature of the body to its proper standard. The kidneys act as a kind of safety-valve, regulating the amount of fluid in the vessels, and allowing more to pass off in proportion as the pressure in the vessels is increased, in consequence of the larger amount ingested, or the diminution of the exhalation by cold, &c. Now in birds, the natural temperature of the body is 10° or 12° above that of the mammalia; and there can be but little need, therefore, of cutaneous exhalation to keep it down. No more fluid, therefore, is required within the body than is requisite for the general purposes of nutrition; and the excretion of the kidneys is voided nearly in a dry state.

were the lightest. From the experiments of Redi it appeared that the duration of life in a fowl totally deprived of food, but supplied with water, was twenty days; whilst those deprived of water also did not live above nine. From a considerable number of observations made on man, it is indubitable that, under ordinary circumstances, a moderate supply of water is favorable to the prolongation of life; and those instances in which the life of man, or of other mammalia, has been prolonged to the greatest extent *without* water, are those in which, from the peculiarity of the circumstances, the cutaneous exhalation must have been reduced to a very small amount, or in which there may have been an actual absorption of water by the skin and lungs. Thus, Fodéré mentions that some workmen were extricated alive, after fourteen days' confinement to a *cold, damp* cavern, in which they had been buried under a ruin. The remarkable case of the hog which was buried in its stye for 160 days, under thirty feet of the chalk of Dover cliff, and was dug out alive at the end of that time, reduced in weight from 160 lbs. to 40 lbs., is well known. In this case the temperature would be kept at a moderate standard by the depth at which the animal was buried; and the air within the small hollow in which it was inclosed would soon become sufficiently charged with moisture to resist any further evaporation. We need scarcely allude to the case recorded by Dr. Willan, in which a young gentleman voluntarily abstained from everything but a little water, just flavored with orange-juice, for upwards of sixty days; in this we have no doubt that the peculiar state of the nervous system exerted an influence (though we do not pretend to assign the mode of its operation,) upon the bodily structure in general, as we see it do in hysterical subjects. In a case of this nature which has fallen under our own observation, and in which we are fully satisfied that there was *no deceit*, there was *complete* abstinence from all solid food during more than three weeks, a small quantity of tea and toast-water being the only fluids ingested, or on some days not even that; and yet, at the end of that period, the flesh was as firm, the voice as strong, and the muscular strength as great, as at the beginning. On two occasions on which, by the entreaty of her friends, the patient was induced to take a morsel of solid food, it was immediately rejected with much violence. The influence of water in prolonging life is particularly shown in reptiles, and especially in the naked-skinned batrachia. A frog, for example, kept without water in a dry atmosphere will lose in a few hours, from the surface of its body, so much water, that its life cannot be prolonged, and it will die of desiccation; but if placed in an atmosphere loaded with dampness, it may be kept alive for a long time, without any supply of liquid.

In order to determine the results of a *forced ingestion* of water, to a larger amount, M. Chossat daily injected a certain quantity into the crops of several turtle-doves, which were totally deprived of food. The result was, that their daily loss of weight was *greater* and the duration of their lives was much *less* than that of birds totally deprived of water. Moreover, they died before they had reached the usual degree of reduction, apparently in consequence of the positively injurious effects of the ingestion of an excessive quantity of water; for it was found, on examination after death, that the blood was more fluid and less disposed to coagulate than in other cases, and that aqueous deposits were present in the lungs, pericardium, &c., impeding the action of the vital organs.

We shall now give a brief sketch of M. Chossat's detailed account of the relative degree of loss sustained by the different organs of the body during complete inanition. The following table presents them in a general form. It will be recollected that the mean or *average* loss of the *whole* body is about ·4; and that of the different organs is set down in the table according as it is above or below this average.

| *Parts which lose* more *than the mean* 0·400 | | *Parts which lose* less *than the mean* 0·400 | |
|---|---|---|---|
| Fat | 0·933 | Muscular portion of the stomach | 0·397 |
| Blood | 0·750 | Pharynx and œsophagus | 0·342 |
| Spleen | 0·714 | Skin | 0·333 |
| Pancreas | 0·641 | Kidneys | 0·319 |
| Liver | 0·520 | Respiratory apparatus | 0·222 |
| Heart | 0·448 | Osseous system | 0·167 |
| Intestines | 0·424 | Eyes | 0·100 |
| Muscles of locomotion | 0·423 | Nervous system | 0·019 |

The almost total loss of the *fat* is precisely what we should expect; knowing, as we do, that this substance is chiefly deposited as a *store*, to be had recourse to in time of need. The reduction in the quantity of blood was measured, not by the simple diminution in its bulk, but by comparison with the amount of blood *of the same quality* in the healthy animal. For the blood of an animal that has died of inanition, is not only much less in bulk, but much poorer in the proportion of its solid contents, than that of a healthy subject; and in order to make the comparison exact, M. Chossat diluted the latter with water, until it exhibited the same amount of attenuation. This seems to us a very inexact mode of comparison; how much better it would have been to have evaporated all the liquid part of the blood, and to have compared the weight of the solid residuum. That the proportional diminution of the blood, however, is far beyond that of the body at large, is evident from this fact,—that in the pigeon, the proportion which the blood bears to the *original* weight of the body is about ·033 in the healthy state, and only ·013 in the inanitiated, whilst the proportion which the blood bears to the *final* weight of the body in the inanitiated animal is ·021. In the rabbit, the proportion which the blood in the vessels at death bears to the *original* weight of the body, is ·0097 to 1; whilst to its final weight it is ·0156 to 1; these proportions, though so different from the preceding, are the same *to each other*, showing that the *relative* diminution in the amount of blood was the same in both instances. The diminution in the size of the spleen is just what we should expect, from our idea of its character as a reservoir or diverticulum of blood; that of the pancreas and liver might be expected to be considerable, from the cessation of the digestive process to which they minister. A very curious circumstance is recorded by M. Chossat, in regard to the state of the blood and liver, in some of the frogs which died after a very prolonged inanition. Scarcely any traces of red blood were found; but it was replaced by a black fluid, resembling diluted cuttle-fish ink, which filled the vessels. The alteration in the character of the liver was so striking, as to give rise to the idea that it was the seat of the change; it had lost its usual colour, and assumed a dark hue, so strong as to leave a permanent spot upon paper, like that made by Indian ink; the bile of the gall-bladder, however, was particularly liquid, and was either green or of a reddish-brown. How much it is to be regretted that a microscopic examination of the state of the blood, and of the tissue of the liver, was not made in these instances! The cruel experiment must now be repeated; before even a probable guess can be hazarded, as to the nature of this curious alteration. On the subject of the proportional diminution in the size of the heart, M. Chossat enters into some curious calculations—the practical use of which, however, does not seem to us to be very evident—in regard to the force of the heart at different ages as compared with that of an inanitiated animal. From these he deduces that, whilst in the normal condition, a heart of 4·17 grammes corresponds in the pigeon with a body of the length of 262 millimeters, and with adult age, a heart of 2·47 granules, (the weight to which this organ is reduced by inanition,) corresponds with a body of 195 millimeters in length, and with an age of only fourteen days; or in other words, the heart of an inanitiated pigeon is no larger than that of a healthy pigeon of fourteen days old. Applying the same results to the human body, he calculates that the heart of man would be reduced by inanition to the size of that of a child of eight or ten years old. The disproportion will probably be still greater, when its relative *powers* is compared, an element left out of consideration by M. Chossat; for it cannot but be supposed that, in two hearts of the same size, the contractile power would be *much greater* in that of the vigorous child than in that of the starved adult. As the muscles of locomotion make up a large part of the bulk of the body in vertebrated animals, and especially in birds, we might anticipate that their reduction would nearly correspond with the average of the whole; and this we see to be the case.

Passing on now to those organs in which the reduction is *less* than the average, we observe that the loss sustained by the muscular portion of the stomach is nearly the same with that of the muscular system in general; but M. Chossat records the curious fact that the thick and horny epithelium which lines the cavity, not only suffers no decrease, but an absolute increase in its weight. This increase is due, however, to an infiltration of fluid; for when dry, the epithelium is not found to have increased, but rather to have diminished, though not in the same proportion with most other parts. A certain degree of softening was observed, resembling an incipient digestion, in the epithelium in the neighbourhood of the cardiac; and pyloric orifices.—The comparatively small diminution in the weight of the lungs and kidneys, is what might have been anticipated, considering that their functional activity as excreting organs undergoes a less diminution than that of most other parts.—The small amount of change in the weight of the *bones*, too, might have been anticipated; knowing, as we do, that they *waste* but little, in comparison with the softer textures. But the most remarkable of all the results supplied by this examination is the maintenance of the normal weight of the nervous centres; the diminution of which was scarcely appreciable in their ordinary state; and but very small when the amount of their solid matter was compared with that of the normal brain and spinal cord, by drying both. Thus amidst the general ruin of the body, the nervous system is kept as nearly as possible in its normal condition, so long as the means of nutrition are supplied to it. We cannot help thinking that the cessation of this supply is often the immediate cause of death. Believing, as we think there is good reason to do, that every act of the nervous system involves a certain disintegration of its substance, and a corresponding demand for reparation, it is easy to see that the suspension of the supply must cause an immediate cessation of its operations; this we take to be the cause of the insensibility in syncope, and in Sir A. Cooper's well-known experiment,—the suspension of the flow of blood to the brain by ligature of the vertebral arteries and pressure on the carotids. The maintenance of the normal weight of the brain, and the general disappearance of the fat, seem to indicate that the former is nourished chiefly at the expense of the latter. The substance of the brain and nerves is well known to be (chemically speaking) an azotized fat; and there are many circumstances which seem to indicate that it is formed, not by a metamorphosis of albumen, but at the expense of fatty matter;* and if this be true, the store of fat laid up in the adipose tissue has a function much more important, than that of merely sustaining the respiration. Future chemical researches may perhaps throw light upon this question.

In proceeding to investigate the influence of inanition upon the amount of heat generated, it was necessary, in the first instance, to ascertain the amount of variation which might take place within the limits of health. From a considerable number of observations, M. Chossat concludes that the temperature of the animal at noon and midnight pretty nearly represents the maximum and minimum, and thus affords the means of readily determining the extreme variation. This last, in the healthy state, may be regarded as about 0°·74 cent., but in the state of inanitiation it averages 3°·28 cent., gradually increasing, from its commencement to its termination. Moreover, the gradual rise of temperature which should present itself between midnight and noon is retarded, and the fall subsequently to noon commences much earlier than in the healthy state; so that the *average* temperature of the body is much lower, even when (as sometimes happens) the maximum temperature remains each day nearly at the natural standard, or is even a little elevated. From the mean of all the experiments it appears, that the average loss of heat between the commencement of the inanitiation and the end of the *penultimate* day of life was about 2°·5 cent.; and dividing this by the average number of days through which life was prolonged, it seems that the average daily loss was no more than 0°·3 cent. But on the *last* day the production of heat diminished very rapidly; and the thermometer fell from hour to hour, until death supervened—the whole loss being, on the average of all the experiments, about 14° cent., or about 47 times as much as on any previous day. It further appeared that the time of death usually corresponded with that of the diurnal depression; for, out of 52 animals experimented on, 36 died between noon and midnight, which is the period of natural diminution, and only 16 between midnight and noon, at which period the temperature is naturally ascending. Perhaps it would have been fairer to compare the twelve hours of the day (from six to six o'clock) with the twelve hours of the night—the whole period of the greatest elevation with the whole period of the greatest depression; and we have little doubt that the result would have been then still more favorable to the doctrine of M. Chossat, who regards the *last* depression as merely a sort of exaggeration of the preceding ones, from which the animal has not power to recover. The *total* average depression at the time of death was about 16·3° cent. or 29·3° Fahr., and the mean temperature of the

* The effect of alcohol in stimulating the nervous system to increased activity seems explicable in some degree by its similarity of composition.

animals at that period was about 76·8°. It is to be regretted, however, that there is no separate statement of the temperature of the birds and the mammalia experimented on, since the difference in their natural standard renders it evidently improper to associate them in such a comparison. In 18 out of 41 cases the temperature at the time of death was between 18·5° cent. (the minimum,) and 24°, or between 65·5° Fahr. and 75°; and in 21 it was between 75° and 86°, in two cases only was it above 86°.

No attempt was made by M. Chossat to determine the relative amount of carbonic acid exhaled at the different periods; but he made observations upon the number of respiratory movements, which show that these diminish in a like proportion. The average number per minute in a state of health was, in the pigeons 31 and in the turtle-doves 49. The mean of the observations between the first and penultimate days gave 23 for the pigeons and 40 for the turtle-doves, and on the last day of life the average was only 19 for the pigeons and 29 for the turtle-doves, the reduction being about 40 per cent. in each. Moreover, it seemed that the actual amount of carbonic acid exhaled did not bear even the usual proportion to the number of respirations; the diminution in the weight of the body being no greater, for some hours before death, than could be accounted for by the evacuations and the cutaneous exhalation; so that it seemed as if the calorifying process had been almost entirely checked for some hours previously. It is remarkable that the number of respirations at midnight should be actually greater, in inanitiated animals, than at midday, though still inferior to the normal standard; or, in other words, the relative diminution is much greater for the diurnal than for the nocturnal respirations. This appears to result from the restlessness which is characteristic of the sleep of animals that are undergoing the process of starvation.

The following are the general symptoms which, according to M. Chossat, presented themselves to observation: In general, the animals remain calm during the first half or two thirds of the period; but they then become more or less agitated, and this state continues for as long as the temperature remains elevated. On the last day of life, however, this restlessness ceases, and gives place to a state of stupor. The animal, when set at liberty, sometimes looks around with astonishment, without attempting to fly, and sometimes closes his eyes, as if in a state of sleep. Gradually the extremities become cold and the limbs so weak as no longer to be able to sustain the animal in the standing posture; it falls over on one side, and remains in any position in which it may be placed, without attempting to move. The respiration becomes slower and slower; the general weakness increases, and the insensibility becomes more profound; the pupil dilates; and life becomes extinct, sometimes in a calm and tranquil manner, sometimes after convulsive actions, producing opisthotonic rigidity of the body.

The observations upon the amount of fecal matter voided by the inanitiated animals are not altogether satisfactory; being exact only as regards their quantity, and taking little account of their quality. After the first day, (in which the fæces contain the residue of the food previously taken,) their amount is very small; and they seem to consist principally of *grass-green biliary matter.* This observation is evidently opposed to

Liebig's doctrine of the reabsorption of the bile ; and is more particularly striking, because if this takes place at all in the manner represented by him, we might imagine it to be especially active, when there is such an *exigeant* demand for the materials of respiration. Towards the close of life, the fæces contain a much larger quantity of water, even when none has been ingested by the animal; and besides the green matter, they are seen to contain white saline matter,—the neglect of the examination of which would have greatly surprised us, if we had not seen in former parts of this Essay, that M. Chossat seems determined to use the *balance* as his only instrument for estimating the condition of the animals experimented on, and regards any departure from his system of weighing, even for the most important purposes, as an injurious interference with his plan. He again recurs to the fact of the duration of life being greater, as the evacuations are less ; but offers no explanation of the variation.

Desirous of testing the correctness of his idea that the cooling of the body is the immediate cause of death, M. Chossat tried the ingenious experiment of placing animals, whose death seemed impending, under the influence of artificial heat ; and the result of this trial was very remarkable. In every instance he delayed subjecting the animals to this influence, until his experience of their state led him to believe that their death must be very near ; and in several cases the animals died whilst he was performing the processes of weighing, &c., preparatory to placing them in the *rechauffoir.* The result was in general to restore those yet alive, from a state of insensibility and want of muscular power, to a condition of comparative activity ; their temperature rose, their muscular power returned, they flew about the room, and took food when it was presented to them ; and, if the artificial assistance was sufficiently prolonged, and they were not again subjected to the starving process, most of them recovered. If they were left to themselves too early, however, the digestive process was not performed, and they ultimately died. Up to the time when they began to take food, their weight continued to diminish ; the secretions being renewed, under the influence of artificial heat, sometimes to a considerable amount. It is not until digestion has actually taken place (which is commonly many hours subsequently to the ingestion of the food), that the animal regains the power of generating heat; up to that period, the heat which its body has acquired from external sources, is lost as soon as ever the supply fails; and thus M. Chossat lost many animals by the accidental cooling of his stove during his absence. This is evidently a point of much practical importance ; and the neglect of sufficient *artificial calorification*, or the too early suspension of it, has doubtless been a frequent cause of the want of success of the means taken to recover inanitiated persons. Several of M. Chossat's birds were lost from the same cause ; and until he arrived at the general principle—that the heat must be artificially sustained, until the quantity of food actually *digested* is fully equal to the wants of the body, he seems to have been unable to account for his results. It is to be remembered that, in these instances, the resources of the body are on the point of being completely exhausted, when the attempt at reanimation is made ; consequently it has nothing whatever to fall back upon ; and the leaving it to itself *at any time* until fresh resources have been provided by it, is consequently as certain a cause of death, as it would have been in the first instance.

In the application of heat to bodies of larger size than the small animals experimented on by M. Chossat, it is to be remembered that a longer time will be necessary for them to become equally affected by it; and means should be taken to apply it more effectually. The warm bath, and, still better, the contact of warm solid bodies with a large part of the surface, should be employed in preference to simple heated air. The same rule applies to the case of children born so prematurely as to require artificial modes of sustaining their heat. In one of the most remarkable of these upon record, it was soon found that no means of applying the warmth were so effectual, as contact with the warm body of another person; and by relays provided for the purpose, this was maintained almost uninterruptedly during the first three weeks of the infant's extra-uterine life. It was observed that, when this was intermitted for the purpose of changing the dress, the child's powers immediately began to flag; although the operation was conducted before a fire, and consequently in an atmosphere at least as hot as its own body.

The memoir concludes with some remarks by M. Chossat on the frequency of *inanitiation* as the real cause of death, in various exhausting diseases. Upon this point we feel much inclined to agree with him; especially since his inquiries upon insufficient alimentation have shown, that this produces effects precisely the same in character with those resulting from complete deprivation of food, though somewhat more tardy in their appearance. It is especially, perhaps, in those forms of febrile disease, in which no decided lesion can be discovered after death, that this view has the strongest claim to reception; and it is here, too, that its practical applications may become most important. For if, as we have good reason to believe, the morbific cause is temporary in its influence, it follows that if we can sustain the system, until it has passed away, the patient who would otherwise have sunk under it may recover. By way of analogy, we may refer to those cases of narcotic poisoning, in which recovery has been due to the artificial maintenance of the respiratory process, during the period when it would have been checked by the narcotism. Now we cannot support the system in fever by *aliment*, for this would not be digested, even if it were taken into the stomach. But we well know the beneficial effects of alcohol in its advanced stages; and the large quantity of this stimulus that may be administered in many cases of fever, is a matter of familiar experience. Now admitting that its beneficial operation is partly due to its specific effect upon the nervous system, we cannot help thinking that we are to regard it as also resulting from the new supply of combustible material, which is thus introduced in the *only* form in which it can be taken up by the vascular system. If we turn our attention for a moment to the state of the digestive apparatus at this period, we shall at once see *why* no other substance should answer the same purpose. In the advanced stage of fever, the secretion of gastric fluid, and the *special absorbent* process which takes place through the villi and lacteals, seem to be in complete abeyance. Still, however, simple *imbibition* may go on through the walls of the blood-vessels, provided that the circumstances are favorable for the production of endosmose; that is, provided the fluid in the alimentary canal is *less dense* than the blood. Now the substances on which we ordinarily depend for the support of the respiratory process, are either of an *oily*, a *saccharine*, or a *mucilaginous* character. Oily substances *cannot* be taken in by imbibition; since they completely check the endosmotic current. Saccharine and mucilaginous substances can only be taken in, when their solution is so dilute, as to be of a density much inferior to that of the blood; hence they must be given in a large bulk of fluid: a practice of which experience has shown the benefit. But alcohol, being already of a density far inferior to that of the blood, is easily absorbed; and, from deficiency of other materials, it is rapidly consumed, so that a very large quantity may be thus ingested, without its stimulating effects being perceptible; just as we see that, in a very cold atmosphere, large quantities of spirituous liquors may be taken with impunity, on account of the rapid combustion they undergo.

Now if there be any truth in these views, there is an obvious deduction from them, of the highest practical importance, viz., that in the advanced stages of fever, when death seems impending, we should endeavour to ward it off by a liberal supply of artificial heat. We have already seen the extraordinary results which this produced upon M. Chossat's starved pigeons and turtle-doves; and we see no reason why similar beneficial results should not present themselves in the case of patients *inanitiated* by fever, though they will be, of course, greatly modified by the morbific cause, so long as it remains in the system. We would earnestly suggest a trial of this expedient, which the simple hot-air bath, now used in many of our hospitals, will readily permit, to those of our readers who may have the opportunity of putting it in practice. We do not *promise* success; but we think that we have shown good physiological grounds, why it may be reasonably expected. We should warn them, however, that the continuance of this external aid for a few hours, or a day or two, is by no means sufficient; but that it must be afforded until the digestive powers are sufficiently re-established, to afford the requisite support to the system through the legitimate channel.

In taking our leave of M. Chossat for the present, we would express our strong sense of the importance of his contribution to experimental physiology; but we should not be discharging our duty to ourselves and to our readers, if we did not at the same time enter our decided protest against the limitation of the scope of his inquiries, which he has assigned to himself. Instead of 'Experimental Researches on Inanition,'—a title which led us to expect a full inquiry into the Morbid Anatomy and Chemistry of the solids and fluids of the animals subjected to experiment, —the heading of this essay should have been 'On the Weight and Temperature of Animals undergoing Starvation.' We fear that M. Chossat was thinking more of the prize to be adjudged by the Academy of Sciences than of the sufferings of the animals he was sacrificing; and whilst we are far from blaming him for undertaking the inquiry, and should by no means quarrel with him for thus restricting the scope of his investigations on any subject that did not involve animal suffering, we *do* blame him for so planning his experiments and observations, that, by the omission of several most important points of inquiry, he has rendered it necessary, for the completion of the history of Inanition, that a similar amount of torture should be again inflicted upon beings that have sensations as acute as our own.

